# The effects of fat-free vs. fat-containing chocolate milk ingestion on muscular strength in female collegiate softball players

**DOI:** 10.1186/1550-2783-9-S1-P3

**Published:** 2012-11-19

**Authors:** Bill Campbell, Ashley Forsyth, Bre Myers, Brittany Parker, Brittany Gomez, Ava Elkins, Colin Wilborn, Paul La Bounty, Brandon Marcello

**Affiliations:** 1 University of South Florida, Tampa, Florida, USA; 2 Mary Hardin-Baylor University, Belton, TX, USA; 3 Baylor University, Waco, Texas, USA; 4 Stanford University, Stanford, California, USA

## Background

Ingesting a post-workout beverage containing carbohydrate and high quality protein has been shown to favorably improve body composition and exercise performance. Chocolate milk supplies both carbohydrate and high quality proteins (casein and whey). For this reason, chocolate milk has become an increasingly popular sports nutrition beverage. To date, no one has investigated the differences between fat-free and fat-containing chocolate milk on strength performance in collegiate athletes. The purpose of this study, therefore, was to determine the effects of ingesting two forms of chocolate milk (fat free vs. fat containing) immediately after resistance exercise over an 8-week period to determine its effects on muscular strength.

## Methods

In a double-blinded manner, 16 female collegiate softball players (18.4 ± 0.6 yrs; 167.1 ± 4.4 cm; 69.5 ± 9.4 kg) were randomized according to strength & bodyweight to ingest a fat free (300 kcals, 58g carbohydrate, 16g protein, 0g fat) or a fat-containing (380 kcals, 58g carbohydrate, 16g protein, 10g fat) chocolate milk beverage. The chocolate milk was ingested in a 16-ounce bottle & occurred immediately following all periodized resistance exercise training sessions for a duration of 8-weeks. Dependent variables included 1RM Bench Press and 1RM Leg Press which were assessed at baseline & following 8-weeks of a periodized resistance training program. Dependent variables were assessed as changes (delta scores) from pre- to post-testing in each group via an independent samples t-test using IBM SPSS Statistics (v19).

## Results

1RM Bench Press at baseline and post-testing for the fat-free milk group was 87.5 ± 18.7 and 98.1 ± 22.8 lbs (an average improvement of 10.6 ± 8.6 pounds). For the fat-containing milk group, 1RM Bench Press at baseline and post-testing was 77.5 ± 11.0 and 90.6 ± 14 lbs (an average improvement of 13.1 ± 6.5 pounds). There were no significant differences in changes from baseline to post-testing between the two groups (p = 0.524). 1RM Leg Press at baseline and post-testing for the fat-free milk group was 285 ± 68.9 and 316.9 ± 94.5 lbs (an average improvement of 31.9 ± 28.3 pounds). For the fat-containing milk group, 1RM Leg Press at baseline and post-testing was 277.5 ± 51.3 and 303.1 ± 51.3 lbs (an average improvement of 25.6 ± 10.5 pounds). There were no significant differences in changes from baseline to post-testing between the two groups (p = 0.567).

## Conclusions

Based on these data, the ingestion of either fat-free chocolate milk or fat-containing chocolate milk will have similar effects in relation to upper and lower body strength changes when ingested immediately following resistance exercise over an 8-week period in collegiate softball players.

